# Supplementary figures and images for: Signatures of hybridization in Trypanosoma brucei
Source: PLoS Pathog. 2022 Feb 9;18(2):e1010300. doi: 10.1371/journal.ppat.1010300 (PMC8863249; doi:10.1371/journal.ppat.1010300)

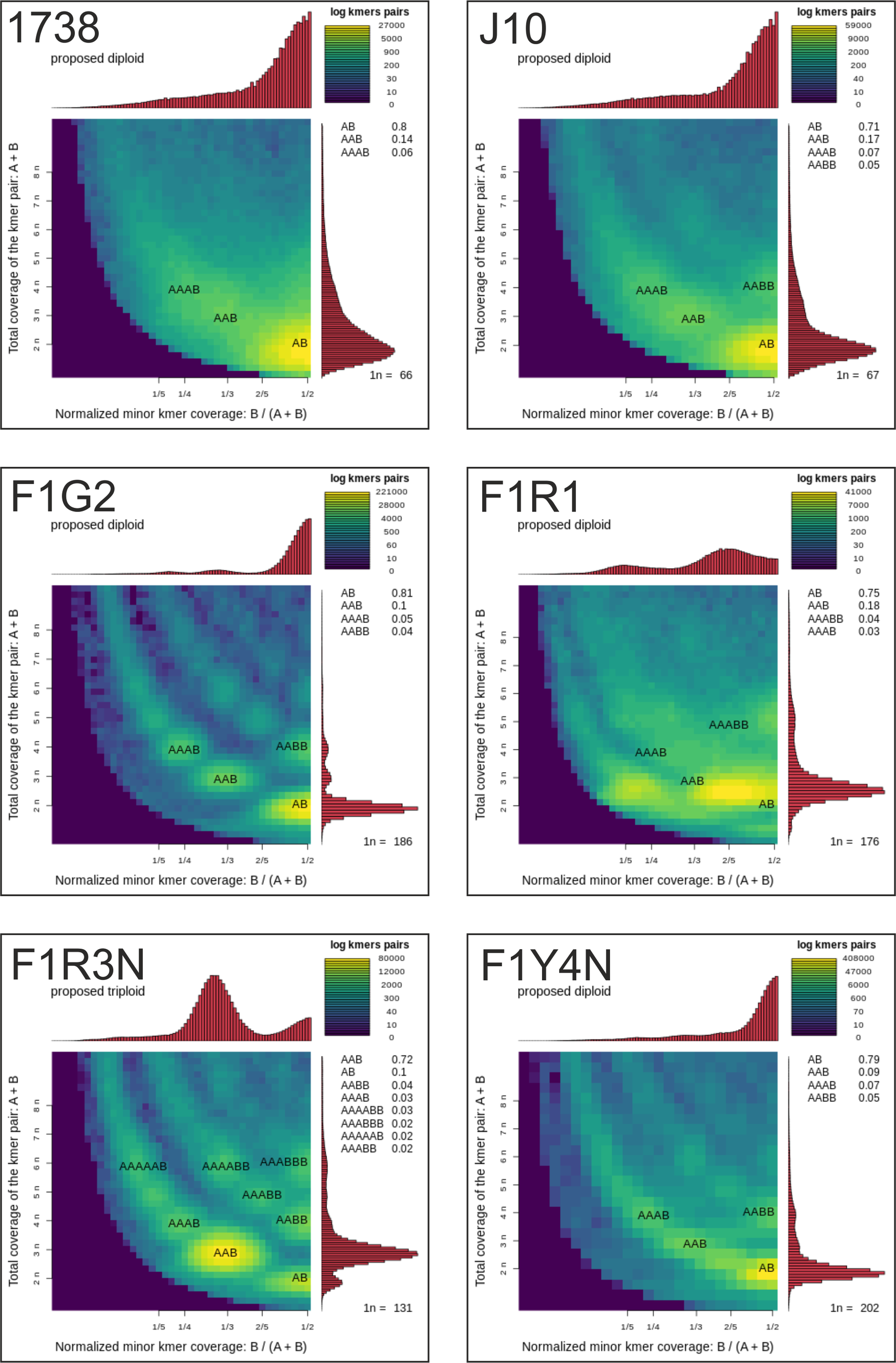

Supplement: S1 Fig — Read pools were quality filtered with Fastp and k-mer histogram tables were then analysed with Smudgeplot (Ranallo-Benavidez TR et al 2020 doi.org/10.1038/s41467-020-14998-3). Proposed ploidy is shown under isolate name, with the probability of other karyotypes shown on the right. Parental strains 1738 and J10, together with hybrid progeny clones F1G2 and F1Y4N, all fit to diploid, while F1R3N fits best to triploid. The anomalous hybrid clone F1R1 has an unusual intermediate pattern inconsistent with a pure diploid population. (TIF) [file ppat.1010300.s001.tif]

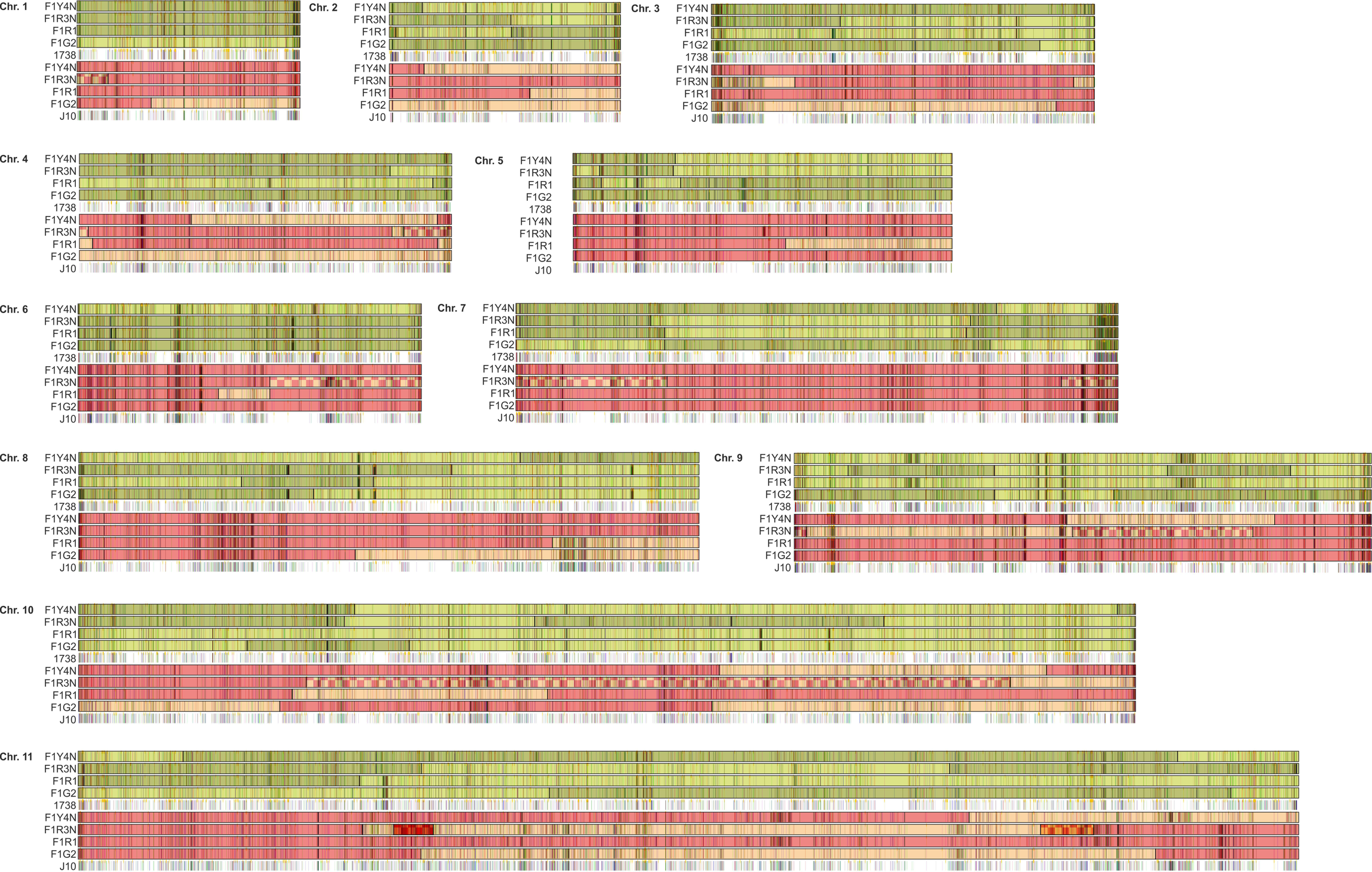

Supplement: S2 Fig — Introgression maps were constructed from patterns of inherited heterozygous SNPs from the parental strains. There were ~20,000 SNPs in each hybrid and the figure illustrates their density and distribution. (TIF) [file ppat.1010300.s002.tif]

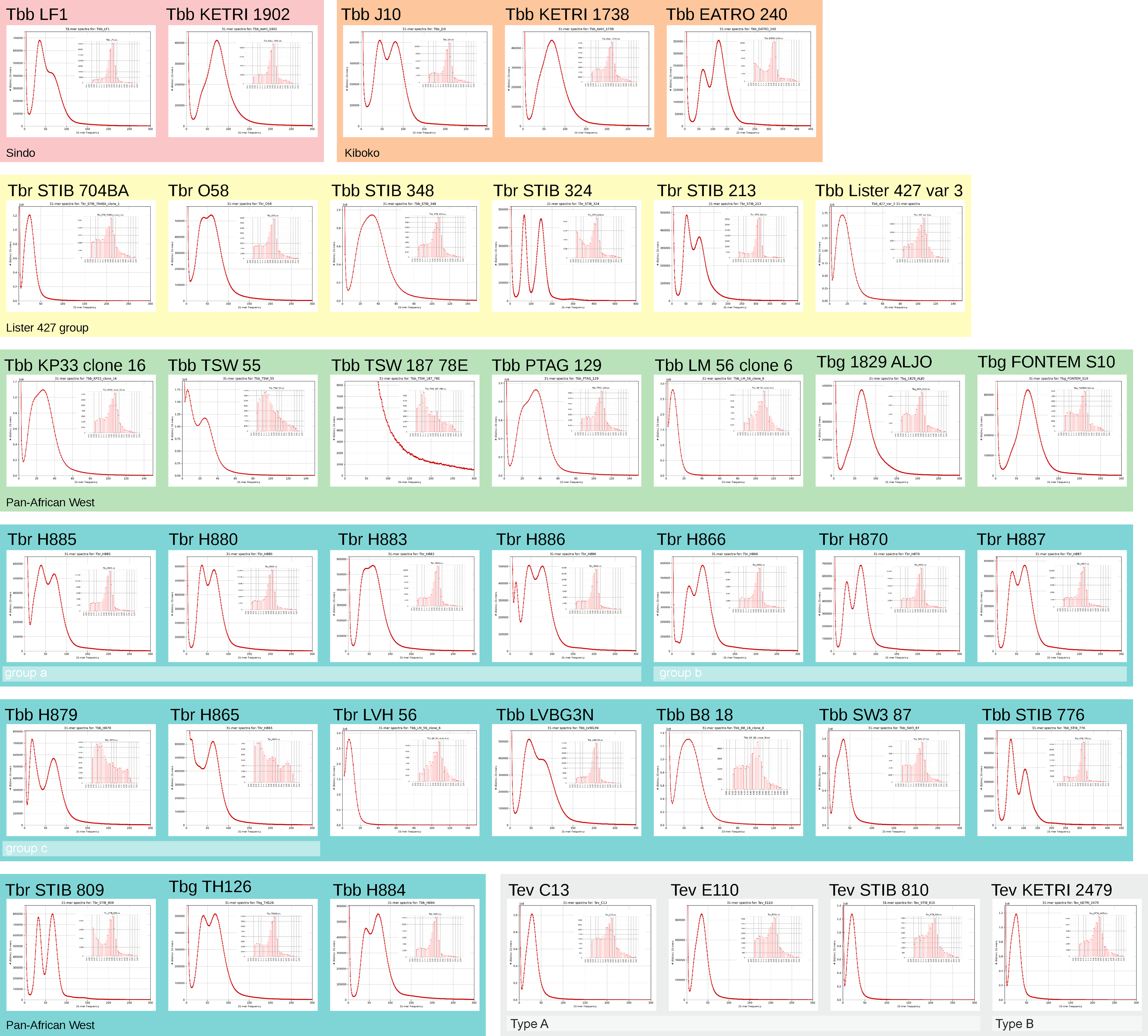

Supplement: S3 Fig — K-mer analysis for the 37 additional field isolates. Although k-mer peaks are unresolved for some isolates, the range of peak shapes suggests that these populations have varying levels of heterozygosity. (TIF) [file ppat.1010300.s003.tif]

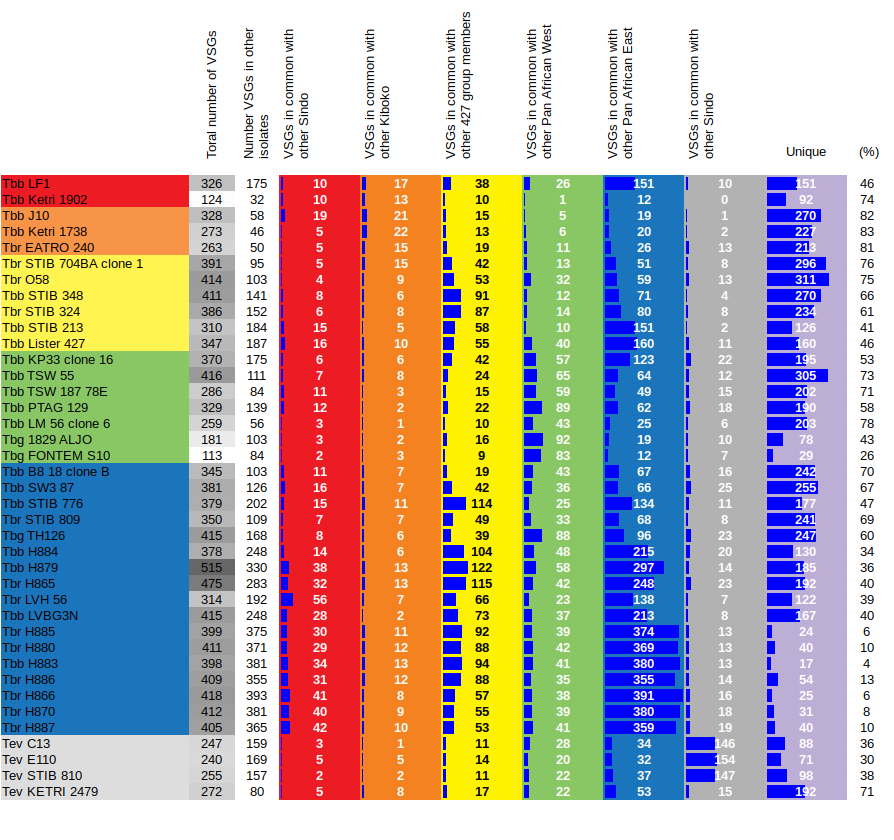

Supplement: S2 Table — (TIF) [file ppat.1010300.s005.tif]
