# Supplementary material for: Signatures of hybridization in Trypanosoma brucei
Source: PLoS Pathog. 2022 Feb 9;18(2):e1010300. doi: 10.1371/journal.ppat.1010300 (PMC8863249; doi:10.1371/journal.ppat.1010300)
Supplement: S1 Text — (DOCX) [file ppat.1010300.s006.docx]

Supplementary methods and data

[**Genome sequencing and assembly** 1](#_Toc87543917)

[***VSG* gene analysis** 4](#_Toc87543918)

[**Metacyclic expression sites** 5](#_Toc87543919)

[**Kinetoplast DNA analysis** 5](#_Toc87543920)

[**Supplementary Data** 10](#_Toc87543921)

[assembled_genomes 10](#_Toc87543922)

[introgression_maps 10](#_Toc87543923)

[Hybrid_and_parent_maxicircle_whole_coding_region 10](#_Toc87543924)

[Hybrid_pooled_minicircles 10](#_Toc87543925)

[Hybrid_VSG_pool 10](#_Toc87543926)

[MES_promoter 10](#_Toc87543927)

[MES_loci 11](#_Toc87543928)

[Maxicircle_sequences 11](#_Toc87543929)

[Sistrom_pooled_minicircles 11](#_Toc87543930)

[Sistrom_VSG_pool 11](#_Toc87543931)

[Tbb_minicircles 11](#_Toc87543932)

[Trypan_glycop_C 11](#_Toc87543933)

### **Genome sequencing and assembly**

DNAs from the four hybrid clones (F1R1, F1G2, F1R3N, F1Y4N) were sequenced by the Earlham Institute, Norwich, UK, using Illumina NovaSeq with 150 bp paired end reads. Illumina sequence data (75 bp reads) from J10 and 1738, together with 37 other subgenus *Trypanozoon* isolates, was kindly provided by Adalgisa Caccone, Yale, USA [1]. Reads were assembled using SPAdes v3.13.1 [2] **(a)**. K-mer frequency analysis was performed using Fastp [3] **(b)**, and histograms produced in KAT [4] **(c)**. SNPs were identified by read alignment using BWA, processed with samtools and bcftools [5] before SNPs were called using Freebayes **(d)**. RTG Tools and Tabix [6] were used to produce numerical reports and a Tablet v1.19.09.03 [7] and Artemis [8] were used to visualise SNP distribution. Further details of genomes are provided in **S1 Table**.

1. For genomic assembly reads were normalised and error corrected using bbtools bbnorm.sh. SPAdes assembly was run with default parameters, though the differences in read length led to assembly finishing at different k-lengths (typically k55 for 75bp read data, k121 for 150bp reads)

**bbnorm.sh in=reads_1.fq in2=reads_2.fq out=corrected_1.fq out2=corrected_2.fq ecc=t target=100 min=3**

**spades.py -o assembly -1 corrected_1.fq -2 corrected_2.fq -t 4 -m 64**

1. Fastp was used to quality filter the read pool

**fastp -i $illumina_1 –I $illumina_2 –o ./1.fq -O ./2.fq -l 55 –q 30**

1. Kat was then used to plot kmer frequency histograms

**kat hist –m31 –t 1.fq 2.fq**

**kat plot spectra-hist kat.hist**

These histogram files were then also used with Smudgeplot [9] to generate **S1 Fig.**

1. Chromosomal introgression, SNP and kmer analysis.

Previously determined core chromosomal regions of *T. brucei* Lister 427 were used to align reads **(i)**. SNPs found in common between parent strains were discarded, as well as homozygous SNPs **(ii)**. The remaining heterozygous SNPs unique to each parent were then used to filter SNPs called in the hybrids. By comparison to each parent, karyotype and introgression events were readily visualised in Artemis (see below). This approach was adapted for SNP calling of the MES locus number 7.

- 1. Read alignment and SNP calling were performed as for kinetoplast (below) except for the substitution for the Tbb Lister 427 chromosomal cores. Ploidy (-p) was fitted to each hybrid, but a –p 2 produced comparable results, the –F parameter was set to 0.2.
  2. The resulting VCF files were compared using vcftools diff command to find SNPs unique to each parent.

Filter by quality:

**vcffilter –f “QUAL > 1500” ./example.vcf**

Remove SNPs common between parents

**vcftools --gzvcf ./SNP_processing/input_filtered/J10_filtered.vcf.gz --gzdiff ./SNP_processing/input_filtered/1738_filtered.vcf.gz --diff-site --out ./SNP_processing/parent_unique_SNPs/differences**

Where the diff file value at the fourth field is used to recover strain specific SNPs, as:

**awk '$4 == "1" ' differences.diff.sites_in_files >> J10**

**awk '$4 == "2" ' differences.diff.sites_in_files >> 1738**

Heterozygous sites are recognised by searching for the allele frequency:

**grep "AF=0.5" J10_unique.vcf >> J10_unique_heterozygous.vcf**

The same process was used to find SNPs common to both hybrid and parent clones and this was done independently for the unique SNPs of each parent. These SNPs were then combined using bcftools concat, producing a final variant call file for each hybrid containing only the SNPs shared with a parental strain.

**bgzip $1**

**bgzip $2**

**tabix -p vcf $1".gz"**

**tabix -p vcf $2".gz"**

**bcftools concat $1".gz" $2".gz" -a -o merged.vcf**

The distribution of SNPs was then visually analyzed in Artemis to produce the introgression maps, an example of this is shown below and a global view is presented in **S2 Fig**.


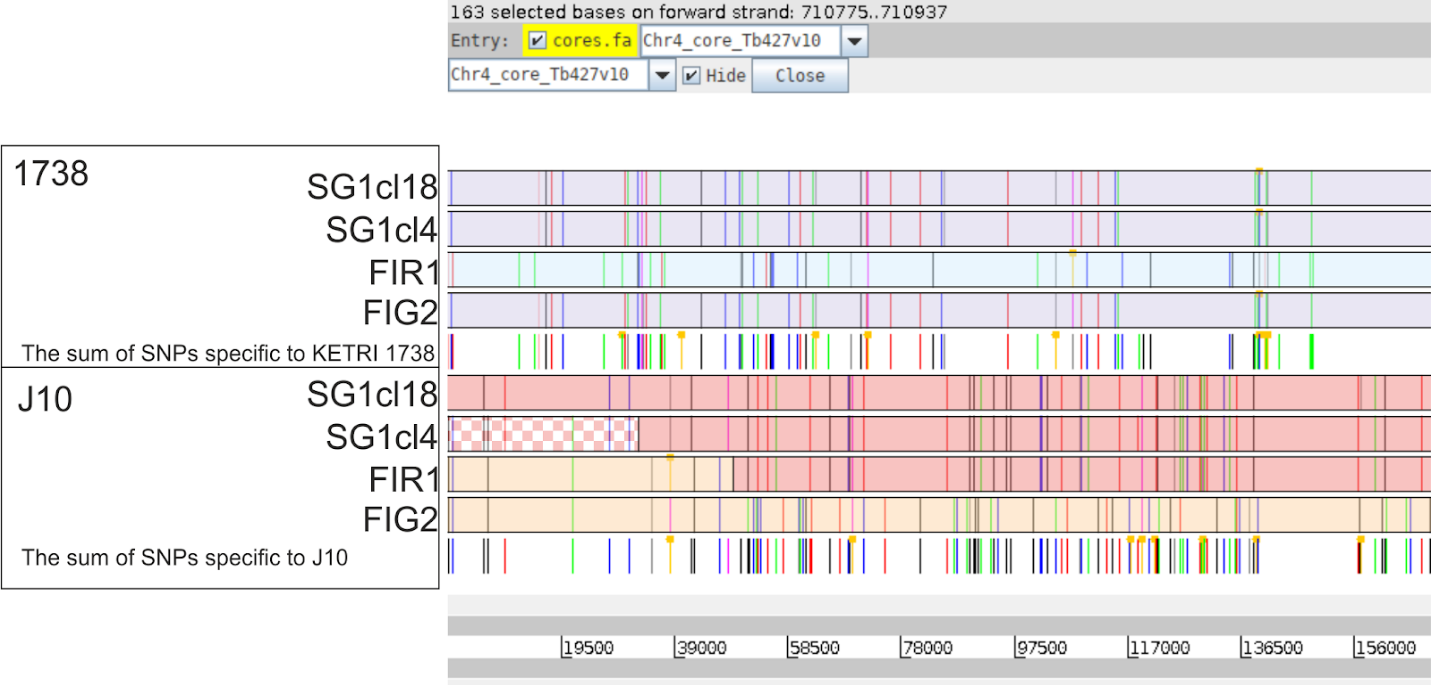


### ***VSG* gene analysis**

*VSG* open reading frames were predicted from the contig pool using Transdecoder [10] **(a)**, and *VSG*s identified by Phmmer [11] **(b)**, using the Pfam HMM for the *VSG* C-terminal domain. For cluster analysis, sequences of greater than 200 amino acids in length with a Phmmer score > 1e-6 were clustered with CD-HIT [12]. Clusters were defined by proteins with regions overlapping 95% of total length sharing 99.5% sequence identity **(c)**.

1. Minimum open reading frame length was 100aa and produced under the default settings of **TransDecoder.LongOrfs**
2. The HMM used to detect VSGs was found at Pfam and included in the supplementary Data. *VSG*s were found using HMMER:
   **hmmsearch --tblout results.txt $hmm $ORFs**

The resulting table was sorted for results of an e value of 1^e-6 or better, these results were then deduplicated before recovering the ORFs and renaming them appropriately.

1. **cd-hit –c 0.995 -s 0.9 -d 100 –l 200**

### **Metacyclic expression sites**

An HMM was derived from known metacyclic promoter sequences[13, 14] and nhmmer [15] was then used to screen genomic contigs with this model. Sequences were clustered by alignment using MAFFT and tree construction via neighbour-joining. These groups were then confirmed by all vs all BLAST; the great variation in contig length and position of overlap made this method preferable over CD-HIT, though largely similar results were obtained. The HMM used to detect the contigs is given in the Supplementary Data, listed at the end of this document, as are the detected loci. Nhmmer was used with default settings, and contigs with evalues greater than e^-6 were used.

### **Kinetoplast DNA analysis**

Minicircles were identified from the contig SPAdes pool using nhmmer [15] with HMM derived from *T. brucei* minicircle sequences from the public Entrez database, using an e-value threshold of 1e-6. Reads aligning to these sequences were extracted from the read pool using Magic-BLAST [16] and a specialised sub-assembly of the reads performed with SPAdes using a kmer value of 55 and the plasmid mode **(a)**. Circular assembled molecules were filtered by size and HMM. Sequences were oriented uniformly by identifying the minicircle conserved sequence blocks by BLAST v2.2.31+ [17] **(b)**. Clustering was performed using CD-HIT [12] with thresholds of >98% identity for sequences within 90% of total length **(c)**. Maxicircles were identified from the contig pool using BLAST v2.2.31+ [17] **(d)**. Sequences were oriented and aligned using MAFFT v7.427 [18] **(e)** and trimmed to the coding region. Site differences were identified using SNP-sites [19]. Heteroplasmy was assessed by aligning the reads to assembled contigs using BWA MEM v0.7.17 [20] **(f)** and visualising the alignment in Tablet v1.19.09.03 [7]. A phylogeny was produced from the alignment using IQ-Tree ModelFinder [83] to compare base substitution models and parameters, and BEAST [21] to perform the final phylogeny **(g)**. Trees were sampled every 1000 iterations over a chain length of 10,000,000; run results were visualised in Tracer [22] and consensus tree by Treeannotator v1.10.4 [21].

1. For minicircle assembly reads were error corrected as before using bbnorm.sh

**bbnorm.sh in=reads_1.fq in2=reads_2.fq out=corrected_1.fq out2=corrected_2.fq ecc=t target=100 min=3**

SPAdes assembly used somewhat different settings to the genomic. In addition to setting the plasmid argument, iterations were limited to k55. Letting SPAdes continue to higher kmer values introduced a short repeat unit on loop closure; whilst small, this impacted our ability to cluster the minicircles subsequently.

**spades.py -o k-55_plasmid -1 corrected_1.fq -2 corrected_2.fq -k 55 –plasmid -t 4 -m 64**

Circular components carried forward for analysis were selected from the SPAdes circular ‘component’ flag.

1. The minicircle HMM used in this analysis is included in the Supplementary Data (listed at end) and was produced by aligning 799 minicircles from Entrez using MAFFT on default settings. The alignment was then trimmed, deduplicated with CD-Hit (-c 0.99) and poor fitting sequences rejected. The remaining 304 sequences were then used to build a HMM using hmmbuild. A skylign plot of the HMM is shown below. This alignment was also used to derive a consensus CSB sequence for minicircle detection (shown left in the skylign figure).


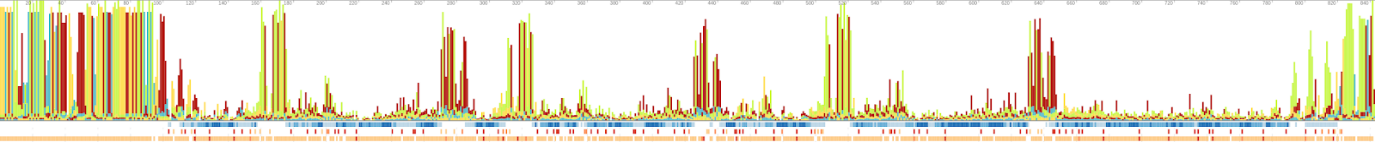


Circular components size filtered from the contig pool (800-1200bp) were first searched for the CSB sequence:

**>CSB**

**TGGGCGTGCAGATTTCACCATACACAAATCCCGTGCTATTTTGGGGCATTTTTGAGGTCCGAGGTACTTCGAAAGGGGTTGGTGTAA**

by BLAST (-perc_identity 85, alignments of greater that 29bp retained), and these results were used to rotate and orient the putative minicircles prior to HMM identification using nhmmer:

**nhmmer --dna --noali --tblout results.txt $hmm $candidates**

1. **cd-hit –c 0.98 -s 0.9 -d 100**
2. For BLAST identification of maxicircles the Tbb Lister 427 maxicircle deposited at Entrez was used as a reference.
3. MAFFT was used with auto settings
4. Reads were first aligned using BWA MEM
   **bwa mem –S –t 4 $maxicircle_file $first_read_file $second_read_file > aligned.sam**

Read alignments were then processed with samtools/bamtools

**samtools view -S -b -F 4 ./get_coverage/aligned.sam > ./get_coverage/aligned.bam**

**samtools sort ./get_coverage/aligned.bam -o ./get_coverage/aligned_sorted.bam**

**samtools faidx $maxicircle_file**

**bamtools index –in aligned_sorted.bam**

Finally SNPs were called with Freebayes

**freebayes –p 1 –F 0.01 -f $maxicircle_file aligned_sorted.bam > freebayes.vcf**

1. A GTR model was chosen with a strict molecular clock, 10% of the sampled trees were discarded as burn-in.

**References**

1. Sistrom M, Evans B, Bjornson R, Gibson W, Balmer O, Mäser P, et al. Comparative genomics reveals multiple genetic backgrounds of human pathogenicity in the *Trypanosoma brucei* complex. Genome Biol Evol. 2014;6(10):2811-9. doi: doi: 10.1093/gbe/evu222.

2. Bankevich A, Nurk S, Antipov D, Gurevich AA, Dvorkin M, Kulikov AS, et al. SPAdes: A new genome assembly algorithm and its applications to single-cell sequencing. Journal of Computational Biology. 2012;19:455-77.

3. Chen S, Zhou Y, Chen Y, Gu J. Fastp: An ultra-fast all-in-one FASTQ preprocessor. Bioinformatics. 2018;34:i884–i90.

4. Mapleson D, Garcia Accinelli G, Kettleborough G, Wright J, Clavijo BJ. KAT: A K-Mer analysis toolkit to quality control NGS datasets and genome assemblies Bioinformatics. 2017;33:574-6. doi: <https://doi.org/10.1093/bioinformatics/btw663>.

5. Li H. A statistical framework for SNP calling, mutation discovery, association mapping and population genetical parameter estimation from sequencing data. Bioinformatics. 2011;27:2987–93.

6. Li H. Tabix: fast retrieval of sequence features from generic TAB-delimited files Bioinformatics. 2011;27:718–9.

7. Milne I, Stephen G, Bayer M, Cock PJ, Pritchard L, Cardle L, et al. Using Tablet for visual exploration of second-generation sequencing data. Briefings in Bioinformatics. 2013;14:193-202. doi: 10.1093/bib/bbs012.

8. Carver T, Harris SR, Berriman M, Parkhill J, McQuillan JA. Artemis: an integrated platform for visualization and analysis of high-throughput sequence-based experimental data. Bioinformatics 2012;28:464–9.

9. Ranallo-Benavidez TR, Jaron KS, Schatz MC. GenomeScope 2.0 and Smudgeplot for reference-free profiling of polyploid genomes. Nature Comms. 2020;11:1432. doi: <https://doi.org/10.1038/s41467-020-14998-3>.

10. Haas BJ, Papanicolaou A, Yassour M, Grabherr M, Blood PD, Bowden J, et al. De novo transcript sequence reconstruction from RNA-seq using the Trinity platform for reference generation and analysis. Nature protocols. 2013;8:1494–512. doi: <https://doi.org/10.1038/nprot.2013.084>.

11. Eddy SR. Accelerated profile HMM searches. PLoS Computational Biology. 2011;7:e1002195.

12. Li W, Jaroszewski L, Godzik A. Clustering of highly homologous sequences to reduce the size of large protein databases. Bioinformatics. 2001;17:282-3. doi: doi: 10.1093/bioinformatics/17.3.282.

13. Kolev NG, Gunzl A, Tschudi C. Metacyclic VSG expression site promoters are recognized by the same general transcription factor that is required for RNA polymerase I transcription of bloodstream expression sites. Mol Biochem Parasitol. 2017;216:52-5. doi: 10.1016/j.molbiopara.2017.07.002. PubMed PMID: WOS:000411771100009.

14. Cross GAM, Kim HS, Wickstead B. Capturing the variant surface glycoprotein repertoire (the VSGnome) of *Trypanosoma brucei* Lister 427. Mol Biochem Parasitol. 2014;195(1):59-73. doi: 10.1016/j.molbiopara.2014.06.004. PubMed PMID: WOS:000341557100010.

15. Wheeler TJ, Eddy SR. nhmmer: DNA homology search with profile HMMs. Bioinformatics. 2013;29:2487-9.

16. Boratyn GM, Thierry-Mieg J, Thierry-Mieg D, Busby B, Madden TL. Magic-BLAST, an accurate RNA-seq aligner for long and short reads. BMC Bioinformatics. 2019;20:405.

17. Altschul SF, Gish W, Miller W, Myers EW, Lipman DJ. Basic local alignment search tool. J Mol Biol. 1990;215:403-10.

18. Katoh K, Standley DM. MAFFT multiple sequence alignment software version 7: improvements in performance and usability. Mol Biol Evol. 2013;30:772-80. doi: 10.1093/molbev/mst010

19. Page AJ, Taylor B, Delaney AJ, Soares J, Seemann T, Keane JA, et al. SNP-sites: rapid efficient extraction of SNPs from multi-FASTA alignments. Microbial Genomics. 2016;2:e000056. doi: 10.1099/mgen.0.000056.

20. Li H, Durbin R. Fast and accurate short read alignment with Burrows-Wheeler transform. Bioinformatics. 2009;25:1754–60. doi: <https://doi.org/10.1093/bioinformatics/btp324>.

21. Bouckaert R, Vaughan TG, Barido-Sottani J, Duchêne S, Fourment M, Gavryushkina A, et al. BEAST 2.5: an advanced software platform for Bayesian evolutionary analysis. PLoS Computational Biology. 2019;15:e1006650. doi: 10.1371/journal.pcbi.1006650.

22. Rambaut A, Drummond AJ, Xie D, Baele G, Suchard MA. Posterior summarization in Bayesian phylogenetics using Tracer 1.7. Systematic Biology. 2018;67:901–4. doi: 10.1093/sysbio/syy032.

### **Supplementary Data**

**Read data for the hybrid clones from the experimental cross is available from the NCBI SRA (Sequence Read Archive,** [**https://www.ncbi.nlm.nih.gov/sra**](https://www.ncbi.nlm.nih.gov/sra) **Project no. PRJNA795331**

**All other data has been deposited in the Dryad repository https://doi.org/10.5061/dryad.xd2547djb. The data package contains the folders listed below. Each folder contains a “Readme” file with further details of the contents.**

#### assembled_genomes

Contains the genomic SPAdes assembled genomes used to predict ORFs and subsequently VSGs, contigs are of the form:
>NODE_166886_length_56_cov_1939465.000000

GGGTTAGGGTTAGGGTTAGGGTTAGGGTTAGGGTTAGGGTTAGGGTTAGGGTTAGG

#### introgression_maps

Contains the VCF files used to construct the introgression maps, the script to derive them, and breakdowns of SNP types / density.

#### Hybrid_and_parent_maxicircle_whole_coding_region

Fasta file containing the commonly oriented and trimmed coding regions of maxicircle contigs

#### Hybrid_pooled_minicircles

Contains the identified contigs from selective subassembly of minicircles. Contig headers inherit their numbering from the SPAdes contigs header (Contig number, contig length, coverage depth)

>Tbb_F1G2#59#1030#370##

GGGCGTGCAGATTTCACCATACACAAATACCGTGCTATTTTCGGGCATTTTTGAGGGCCGTGGTACTTCGAAAGGGG

#### Hybrid_VSG_pool

Contains a fasta of identified VSGs, entry headers include information about their origin. # separated fields indicate, isolate, SPAdes contig, contig length, coverage depth, position/orientation of ORF, HMM used, and HMM evalue

>Tbb_F1G2_k-121#1576#5315#45#1836-3419(+)#Trypan_glycop_C#8e-29

MLKKLALTAIVLAFSNGRKATGAALNDGDNAKYFKPLCGIIRAASAAPEAVPEQPVLDDLEATALLINLSYASPKAMSELTA

#### MES_promoter

Is the hmm used to identify MES contigs.

#### MES_loci

Presented as a fasta in the form:

>Cluster_1#Tbb_FIG2#114#31951#R

CCAGACACCCTTAGAGACAGAGGGGGTATGCAATCAACAAGCAACAGAAACAGAAAGAGGGGAAGAGAATAATA

Where contig, isolate, genomic SPAdes assembly contig number, length, and orientation relative to assembly (R indicates the sequence has been reversed)

#### Maxicircle_sequences

Identified maxicircle contigs from the field isolates.

#### Sistrom_pooled_minicircles

As for the minicircles fasta above, except includes other isolates, and does not include hybrids

#### Sistrom_VSG_pool

As for the VSG fasta above, except includes other isolates, and does not include hybrids

#### Tbb_minicircles

Is the derived hmm from our assembled minicircles

#### Trypan_glycop_C

Is the PFAM hmm for the VSG C terminal domain at the time of submission
