# Supplementary material for: Signatures of hybridization in Trypanosoma brucei
Source: PLoS Pathog. 2022 Feb 9;18(2):e1010300. doi: 10.1371/journal.ppat.1010300 (PMC8863249; doi:10.1371/journal.ppat.1010300)
Supplement: Attachment — Submitted filename: Response_to_reviews_PloSPath_v2.docx [file ppat.1010300.s007.docx]

**Response to reviewers "Signatures of hybridization in Trypanosoma brucei"**

Many thanks to the editorial team and reviewers for evaluation of this paper. As reviewer 2 is largely complimentary – thanks for the kind words! – most of this response addresses the comments of reviewer 1. Reviews are copied below, with points to be addressed in italics.

**Reviewer #1**:

Kay et al. address the question of genetic exchange in Trypanosoma brucei (and closely related species) using data from whole genome sequencing and assembly. Some of the sequencing data are new to this study, most are from previously published work, but all of the analyses are novel. Using data from lab hybrids, the authors present evidence of chromosomal cross-overs and post-meiotic ploidy change that have important implications for the largely cryptic mechanisms of genetic exchange in this species. They confirm previously proposed patterns of kDNA inheritance. These are then used in an analysis of VSG gene and kDNA maxi/minicircle composition in lab hybrids and field isolates, concluding that there is evidence of genetic exchange in these data even between relatively distantly related strains.

The mechanism of genetic exchange in T. brucei is an important and active topic and I found the addition of support for an emerging model of meiosis very interesting. The findings on kDNA and VSG inheritance are largely confirmatory, but I think constitute the best evidence to date. Similarly, there is existing support for genetic exchange (at least in T. brucei brucei and T. brucei rhodesiense), but additional support would be an important finding. However, sequencing depth, quality and assembly create a substantial source of potential artefact to the analysis and I don’t think this is currently adequately addressed. This particularly affects the analysis of VSGs, although it also has an expected influence on k-mer and SNP analysis. As such, in my opinion the data presented don’t currently support the major conclusions of the manuscript.

**Part II – Major Issues: Key Experiments Required for Acceptance**

*1) Analysis of lab hybrids
I don’t think the distribution of K-mers in F1R1 supports the authors’ interpretation of selfing. At least, I can find no proportion of Pop A and Pop B that would produce the distribution shown. If selfing is the cause, then the k-mer peak from additional genes in Pop B and the k-mer peak from genes not present in Pop B should be symmetrical around the 1x peak (with the shift in both directions dependent on the proportion of B). Isn’t it more likely that this distribution represents a mix of 2 clones or the presence of contaminating DNA in the sequencing? If the authors want to conclude that this is selfing, I think they would need to show that a ratio of A/B exists that can produce the distribution observed (I was unable to find one) and also rule out other easier explanations. For all of the data in Fig 1, it would be really useful for the inferred integer copy numbers be put on the distributions, so the match/mismatch to expectation can be assessed.*

Indeed, the F1R1 k-mer and SNP results were very puzzling and we have thoroughly re-evaluated them in the light of the reviewer’s comments. Further explanation has been included in the text to explain why we ruled out contamination with another trypanosome strain or hybrid clone, leaving selfing as the logical conclusion. The text has also been altered to emphasize the uncertainties in interpretation and we revised the F1R1 panel in Fig 1d and legend accordingly. New text:

“Surprisingly, the other diploid hybrid clone (F1R1) did not conform to this pattern, with both the k-mer and SNP frequency plots showing multiple peaks (**Fig 1d**), suggesting that this “clone” consisted of a mixture of trypanosome strains. However, none of our subsequent analyses (see below) revealed the presence of nuclear or kinetoplast DNA from any strain outside the J10 x 1738 cross, and a mixture of two different hybrid clones would have produced anomalously large repertoires of *VSG* genes and kDNA minicircles. Therefore, our working hypothesis is that only genetic material from F1R1 is present, but that it has been reorganised through selfing. Of note is that the DNA used for genome sequencing here was derived after fly transmission of the original F1R1 clone without re-cloning, on the assumption that clones are transmitted faithfully because selfing only happens infrequently [34-36]. The complexity of the k-mer and SNP frequency plots (**Fig 1d**) suggests that a mixture of F1R1 and a selfed population is present. However, mixtures of F1R1 with a diploid selfed population would give rise to SNP peaks at approximately 0.25, 0.5 and 0.75, whereas the observed peaks are at approximately 0.2, 0.4, 0.6 and 0.8 (**Fig 1d**); in particular, the lack of the 0.5 peak does not fit with any mixture containing a diploid selfed population, suggesting instead that a triploid selfed population is present, and interestingly this hypothesis is supported by the Smudgeplot [37] ploidy analysis (**S1 Fig**).

Despite the uncertainties in interpretation, we kept F1R1 in the analysis,…”

To support our overall interpretation of ploidy in the hybrid clones, we carried out complementary analysis using Smudgeplot, which has been included as a supplementary figure (S1 Fig), with methods added to the supplementary file, S1 File.

*I also didn’t understand the SNP frequency histograms. Heterozygous alleles give SNP frequency modes at 0.5 (as per parents and F1G2 and F1Y4N). Alleles with greater ploidy should show peaks at rational frequencies (e.g. 1/3 and 2/3 for triploids, 1/4, 2/4, 3/4 for tetraploids, etc.). Again, the existence of irrational SNP frequency modes in F1R1 seems to me to be evidence against the author’s conclusion that this is selfing (which would still give rational modes). The authors attribute the lowest SNP frequency modes to the lowest k-mer modes, which doesn’t seem to make sense. How can a monoallelic gene have a SNP frequency? Why would 2x genes contribute to a peak in SNPs not at 0.5? Wouldn’t it make more sense for both peaks at 1/3 and 2/3 in F1R3N to come from the 3x genes? There is something about these data that I am not understanding as presented.*

Our SNP analysis considered only the mapped chromosomal cores, not the subtelomeres and their associated single copy genes, so there should not be any monoallelic genes. The SNP profiles generated match those produced by Tihon et al (Mol Ecol 2017 10.1111/mec.14271) (Fig 1) for diploid and triploid strains. Furthermore, synthetically mixed/contaminated samples in the Tihon et al. paper generated characteristic sloping profiles not observed here for any of our samples. We also tried a simulation of a contaminated read pool containing reads from two hybrid clones, as shown below:

In this scenario, the *VSG* repertoire is anomalously large, the Smudgeplot falters on calling ploidy, while the k-mer and SNP frequency plots do not resemble those of F1R1. Increasing the proportion of reads from the contaminating clone is likely to further increase the number of *VSG*s. We conclude that if other hybrids, or another strain were present (a ‘contaminated sample’) we would have more *VSG*s than we can account for. Ruling out this scenario, the complexity in the F1R1 plots must originate from a common pool of genetic material, where the alleles exist at different copy numbers, i.e. there is a mixed population, but of the original F1R1 clone with another selfed population. Intriguingly, the Smudgeplot (S1 Fig) suggests a mixture of diploid and triploid organisms, and this scenario would yield the observed SNP frequency peaks as shown below, given that chromosomes in the triploid clone (population B) have undergone meiotic crossing-over.

*“J10 apparently having greater heterozygosity than 1738, as it has a more prominent haploid peak in the kmer plot, and more heterozygous sites in the SNP plot”. Firstly, the haploid peak for 1738 is obscured by the overlapping diploid peak, so the size cannot be assessed without fitting. More importantly, if the read depth or sequencing length/quality were considerably lower for 1738 than J10 (as one might infer from the peak spreads in Fig 1) this could greatly affect both the chances of assembly of haploid areas of the genome and the calling of SNPs (versus sequencing errors). This will have knock-on affects for both VSG set analysis and introgression analysis. Note that this won’t have the same affect on all parts of the genome, so unfortunately I don’t think metrics such as N50 or total assembled sequence will adequately account for possible artefact, here.*

Agreed and this sentence has been revised: “J10 apparently having greater heterozygosity than 1738, as it has more heterozygous sites in the SNP plot”. While the k-mer plots are different, we could find no additional evidence that the sequence data from J10 and 1738 are substantially different.

*There is a big jump in analysis to produce the schematics in Fig 2, but the data used to arrive at these conclusions are not presented and no indication of confidence is provided. These are important inferences for the work, so some attempt needs to be made to show readers how these conclusions have been reached. How have calls been made about when a block is from one of other parent or mixed? How many SNPs of what confidence support each crossover event? There are established tests for detecting introgression (e.g. the D- and S*-statistics), but these don’t appear to have been applied here. At the least, it would be really useful to see a map of the density and classification of SNPs across the chromosomes in Supplement and a fuller explanation of how this has been parsed to produce Fig 2.*

This has now been provided as a supplementary figure (S2 Fig).

*2) Analysis of VSG content and comparison to phylogeny
The authors need to be clearer that VSG numbers reflect assembly and detection not just representation in the genomes. Unless these lines have an order of magnitude fewer VSG genes than the genomes of lines mostly studied in the lab, then all of these sets are only a very small proportion of the full content. “Each clone had inherited approximately half its VSGs from each parent, with a bias to inheritance from J10 (paired t test, p = 0.0335, 95% CI), perhaps because J10 had more VSGs than 1738.” [line 258] suffers from the same artefact as the k-mer analysis, and it needs to be clear that “perhaps because we were able to assemble fewer VSG from this 1738” is likely more accurate. Similar for “Inheritance of these arrays in individual hybrid clones follows the same pattern” [line 277].

For this reason, in my opinion it is vital that shared VSG content (in analysis of hybrids and field isolates) be expressed as some measure of set-similarity, not as absolute numbers. For example, sharing only 12 VSG could either be a very low amount of overlap, or mean that every single VSG of 12 detected in one strain was also found in another strain. This is a particular issue when it comes to the analysis of “distantly related lineages” (Figs. 5 and 6). The analysis of linkages is expected to be substantially influenced by the completeness of the VSG/minicircle assembly. Indeed, all of the links highlighted in the text are between strains with some of the greatest number of assembled VSG/minicircles and I think a more convincing analysis is needed (ideally with some statistical support) to demonstrate this is significantly more than can be explained by assembly completeness.*

We have added text to clarify why the total number of *VSG*s found here is smaller than the widely accepted 1000 *VSG* ballpark figure.

“The total number of VSGs per genome found here falls far short of the typical 1000 VSG estimate derived from DNA hybridization densitometry or genome assembly [39, 43], since these totals include closely similar genes, which would be clustered here, and pseudogenes, whereas only open reading frames were counted here.”

Here, it was meaningful to consider only the set of individual *VSG*s where sequence differences allowed inheritance to be followed unequivocally from parents to progeny. Though the numbers of *VSG*s recovered from field isolates do not reflect their full *VSG* repertoires, nevertheless they still furnish hundreds of markers to study field populations.

We have modified the text (line 258 now 283) as suggested:

“Each clone had inherited approximately half its *VSG*s from each parent, with a bias to inheritance from J10 (paired t test, p = 0.0335), perhaps because J10 had more *VSG*s than 1738, or that the assembly of 1738 was less contiguous/complete.”

The number of assembled *VSG*s did not correlate to the amount of read data, as shown in the analysis below. Even the superior depth of sequencing of the hybrid clones did not privilege them with respect to *VSG* number. This is further evidence that we are reliably capturing all the *VSG*s that meet our detection criteria, even at the lowest read depths.

*The finding of discordance between kDNA maxicircle phylogeny and VSG/minicircle content for 2 isolates H865, H879 is really interesting, and suggestive. However, the Mx1 clade has a posterior probability of only 0.34 representing really weak support in this area of the tree and there is no confidence at all given for the clustering of H865 and H879 into type ‘c’ VSG/minicircle content. Note that both of these classifications need to be well-supported for the hypothesis of hybrid origin to have support, so proposing that this represents “compelling evidence” is a considerable over-reach. Either additional support needs to be provided, or the conclusions with respect to these data need to be substantially revised.*

Indeed, this clade does have weak support, and we have now pointed this out in the text (“with the two isolates in cluster c subsumed into either Mx1 (H865) or Mx2 (H879), though statistical support for the exact position of H865 in Mx1 is low”) and replaced “compelling evidence” with “the evidence suggests” in the concluding paragraph.

**Part III – Minor Issues: Editorial and Data Presentation Modifications**

*In the reference genomes, there are quite a few VSG in the chromosome cores. How many of the genes in the analysis of hybrids are from core regions?*
As noted above, the approach used here to identify *VSG*s was different to the assembly and annotation of *VSG*s in reference genomes, so we are not able to determine how many of the *VSG*s are in core regions of the 11 large chromosomes, in telomeric positions or on smaller chromosomes.

*How can the parental lines contain “Additional VSGs not found in either parent” as represented by white circles in Fig 3?*
The legend to Fig 3 has been modified; this part of the legend refers to the lower panels showing hybrids, while the top panel shows the parents.

*What does “95% CI” indicate in the reporting of statistical tests? A confidence interval would usually be placed on an estimate of a value. Is the meaning here that the authors have chosen α<0.05 as the threshold for significance? I would suggest that reporting of the p-value is sufficient.*
Indeed, “95% CI” was unnecessary and has been removed.

*“**The longest contig in each cluster terminated downstream of the promoter in telomeric repeats (TTAGGG) and/or a VSG (with the exception of cluster 6)” [line 290]. Is really unclear as written. Are contigs with just a promoter and telomeric repeat being considered MES here? This doesn’t seem right.*
This has been rewritten:

“The longest contig in each cluster terminated downstream of the promoter and *VSG* in telomeric repeats (TTAGGG) (with the exception of cluster 6)”. We have also added a reference to our recent paper on BioRxiv that gives expression data on these *mVSG*s in trypanosomes derived from the tsetse salivary glands. https://www.biorxiv.org/content/10.1101/2021.10.13.463681v2

*I found having the strains presented in a different order in Fig.5a and 5b unhelpful and counter to the point of the figure, which is to compare phylogeny with VSG/minicircle content. Could the data be reordered or a tanglegram be included to aid navigation?*
Figure 5 has been modified accordingly.

*I didn’t find that Fig 6 added very much to understanding, as it mostly represents a subset of the analysis presented in Fig 5. I would recommend it is moved to the Supplement.*

We would like to retain the circos diagram as it complements the data shown in Fig 5 in an accessible and visually appealing way.

**Reviewer #2:** The manuscript by Kay et al presents an in-depth genomic analysis of several T. brucei isolates, including those from a genetic cross (parentals + descendents + siblings), which gave the authors important information on how recombination and gamete fusion occurs in the fly, as observed by the genomic evidence, and how this is correlated with the observed outcomes (diploids, triploids).

The manuscript is well written, complete and providing very important data for the community of scientists working in kinetoplastids. Important information is provided on how kDNA is passed onto progeny after a cross or through hybridization. Also very important information is provided on how VSGs, and MVSGs are shuffled in a cross.

Also very important information arises from use of all these observations on genetic hallmarks of recombination, and inheritance to field isolates from different outbreaks, and along large time spans (years).

I'd say this is a landmark paper that will guide the field for a number of years to come.

**Part II – Major Issues: Key Experiments Required for Acceptance**

I congratulate the authors as I've have a very hard time finding _any_ suggestion to make, or a typo or mistake to highlight.

*The only major issue in this manuscript is the lack of accession numbers for all the sequenced genomes. The "Availability of data and material" reads "All data generated or analysed during this study are included in this published article and its supplementary information files." However the raw FASTQ files were not part of the supplementary materials and I failed to see accession numbers for these data mentioned in the Table S1, or S1 File. This must be corrected before publishing.*

Indeed, our intention was to put all this material online if the paper was accepted. All the assembled analysis data (VCF files, minicircles, etc.) will be made publicly available via Dryad, and sequencing data via SRA. A list of this data is given at the end of Supplementary Methods (S1 File).

**Part III – Minor Issues: Editorial and Data Presentation Modifications**

I have not identified any minor issue in this manuscript. Congrats to the authors!
Perhaps my only suggestion would be *to include a graphic or schematic to summarize the observations on how nuclear, maxicircles, minicircles, VSGs, MVSGs are inherited after a cross, hybridization, etc. And which scenarios would produce different ploidies.* I think this will help wrap up the manuscript coming full circle and presenting the readership an overview or guide to use genomic data to interpret these different scenarios (e.g. when sequencing a new isolate and trying to guess the clonal descent from a reference, or identifying putative parentals in extant sequenced genomes). This could be used also as a graphical abstract maybe.

Thanks for the suggestion. We have included a new figure 7.
