# Supplementary material for: Signatures of hybridization in Trypanosoma brucei
Source: PLoS Pathog. 2022 Feb 9;18(2):e1010300. doi: 10.1371/journal.ppat.1010300 (PMC8863249; doi:10.1371/journal.ppat.1010300)
Supplement: Attachment — Submitted filename: Response to reviewers_revision.docx [file ppat.1010300.s008.docx]

**Response to reviewers "Signatures of hybridization in Trypanosoma brucei"**

Reviewer's Responses to Questions

**Part I - Summary**
Reviewer #1: Thanks to the authors for engaging with my comments, particularly in the light of divergent opinion between the 2 reviewers.

F1R1 k-mer and SNP results
It was good to see that on re-analysis the authors agree that the previous hypothesis (mixture of 2 diploids with selfing) didn’t fit the data. I found the new analysis really helpful in seeing how the authors had parsed the data and the sequencing mixing and smudgeplot analyses are good additions. I’m still not convinced that a mix of diploid and triploid populations represents the only viable interpretation of these data (wouldn’t a single population in which only a single/few chromosomes are triploid also fit?), but the revised text doesn’t claim this as the only possibility and the data certainly seem compatible with such a situation.

SNP frequency histograms
I don’t want to be difficult here, and I apologize in advance if this is just a lack of understanding on my part, but the interpretation of the SNP histograms in Fig.1 still doesn’t make sense to me. The authors begin their rebuttal of my concern “Our SNP analysis considered only the mapped chromosomal cores [...] so there should not be any monoallelic genes”. However, their interpretation of the k-mer peaks in Fig.1 explicitly requires the existence of monoallelic genes which are marked red in all of the panels (a-f) and marked ‘1x’ in a,b,c and e. The figure still seems to mis-assign the SNP frequency peaks to the gene copy numbers. For example, F1R3N (e) shows data from a triploid strain expected to contain 1x copy genes (e.g. VSG), 2x (fewer as per lower k-mer peak) and 3x genes (blue dot in fig) as per the authors’ schematic. Haploid (1x) genes don’t have homologs in the same strain, so any read differences are just down to sequencing errors, which should to my understanding give ‘SNP frequency’ peaks near 0 or 1 (i.e. outside of the display range). Diploid genes can either be homozygous for a SNP (again 0 or 1) or heterozygous resulting in peak at 0.5. This peak appears to be too low to be detected in Fig. 1e. Triploid genes could again be homozygous at a SNP position (0 or 1) or 2:1 heterozygous (peaks at 1/3 and 2/3) or 1:1:1 heterozygous (peak at 1/3). The authors instead attribute the peak at 1/3 to the 1x genes and that at 2/3 to 2x. This still seems incorrect. It is possible I am badly misinterpreting these data, but I’m afraid the rebuttal didn’t clear this up for me.
Apologies for the confusion. On re-examining the figure, it’s clear that the colour assignment of peaks has gone awry. The colours in panels d and e have now been changed, so that the alleles in the chromosome cartoons match the SNP peak assignments.

Analysis of VSG content
Please note that my concern here was from the influence of completeness of VSG /assembly/ not read depth. While read depth could be a contributor to incompleteness it is not the only factor, so while the comparison of number of reads and number of VSGs was welcome it doesn’t really address my concern here. I am still of the opinion that analysis of the set-similarity rather than absolute numbers of VSG would be a much more suitable method here and offset many concerns about the influence of assembly completeness. However, I note that Reviewer 2 did not match any of my concerns and the authors have helpfully modified some of the text around these data, so I feel it is the authors’ decision whether or not to act here.
Thanks again for taking the time to consider the results. As this is not a major point of contention, we have left the analysis as is.

Minor points
All minor points raised have been addressed.

**Part II – Major Issues: Key Experiments Required for Acceptance**
Please use this section to detail the key new experiments or modifications of existing experiments that should be absolutely required to validate study conclusions.

Generally, there should be no more than 3 such required experiments or major modifications for a "Major Revision" recommendation. If more than 3 experiments are necessary to validate the study conclusions, then you are encouraged to recommend "Reject".

Reviewer #1: I am of the opinion that the SNP frequency histograms (see above) still require correction or explanation before acceptance.

As noted above, Fig1 has been revised.

**Part III – Minor Issues: Editorial and Data Presentation Modifications**
Please use this section for editorial suggestions as well as relatively minor modifications of existing data that would enhance clarity.

Reviewer #1: All minor points raised have been addressed.
